# Retreating or Standing: Responses of Forest Species and Steppe Species to Climate Change in Arid Eastern Central Asia

**DOI:** 10.1371/journal.pone.0061954

**Published:** 2013-04-15

**Authors:** Hong-Xiang Zhang, Ming-Li Zhang, Stewart C. Sanderson

**Affiliations:** 1 Key Laboratory of Biogeography and Bioresource in Arid Land, Xinjiang Institute of Ecology and Geography, Chinese Academy of Sciences, Urumqi, China; 2 University of Chinese Academy of Sciences, Beijing, China; 3 Institute of Botany, Chinese Academy of Sciences, Beijing, China; 4 Shrub Sciences Laboratory, Intermountain Research Station, Forest Service, United States Department of Agriculture, Provo, Utah, United States of America; University of Florence, Italy

## Abstract

**Background:**

The temperature in arid Eastern Central Asia is projected to increase in the future, accompanied by increased variability of precipitation. To investigate the impacts of climate change on plant species in this area, we selected two widespread species as candidates, *Clematis sibirica* and *C. songorica,* from montane coniferous forest and arid steppe habitats respectively.

**Methodology/Principal Findings:**

We employed a combined approach of molecular phylogeography and species distribution modelling (SDM) to predict the future responses of these two species to climate change, utilizing evidence of responses from the past. Genetic data for *C. sibirica* shows a significant phylogeographical signal (*N*_ST_ > *F*_ST_, *P*<0.05) and demographic contraction during the glacial-interglacial cycles in the Pleistocene. This forest species would likely experience range reduction, though without genetic loss, in the face of future climate change. In contrast, SDMs predict that *C. songorica*, a steppe species, should maintain a consistently stable potential distribution under the Last Glacial Maximum (LGM) and the future climatic conditions referring to its existing potential distribution. Molecular results indicate that the presence of significant phylogeographical signal in this steppe species is rejected and this species contains a high level of genetic differentiation among populations in cpDNA, likely benefiting from stable habitats over a lengthy time period.

**Conclusions/Significance:**

Evidence from the molecular phylogeography of these two species, the forest species is more sensitive to past climate changes than the steppe species. SDMs predict that the forest species will face the challenge of potential range contraction in the future more than the steppe species. This provides a perspective on ecological management in arid Eastern Central Asia, indicating that increased attention should be paid to montane forest species, due to their high sensitivity to disturbance.

## Introduction

Addressing the impacts of climate change on the distribution and genetic structure of species has become an active field of research in historical biogeography [1] and global change biology [2], [3]. One approach that may be fruitful is the employment of responses to past climatic change, especially during the Pleistocene climatic oscillations, in order to predict effects from expected changes in the future [4], [5]. Past climatic change has profoundly shaped present species distributions and spatial genetic structure [6], [7], [8], and in the future it is predicted to accelerate biodiversity loss and threaten current distributions of species [2], [9].

Climatic changes have affected species distributions and their lineage evolution in many parts of the world. Species in high-latitude regions are highly sensitive to global climate change [10], [11], [12]. As evidenced from fossil records and spatial genetic structure, many northern species experienced southward migrations and divergence among lineages that survived in separate refugia during cold glacial periods [10], [11]. Facing recent global warming, species in northern high-latitude regions are threatened with northward retreat and loss of genetic diversity [5], [12], [13]. In tropical regions, climate changes have also left their footprints, which influence species distribution patterns and drive species genetic divergences [14], [15], [16].

As one of the more widespread landscape types in the world, arid lands are also known to have experienced a series of climate changes during the Quaternary [17], [18], which have strongly influenced the distribution and evolution of organisms [19], [20], [21]. In the future, climate changes are similarly projected to occur in arid lands [22], [23], but information is needed on expected range shifts and genetic responses of plant species to these changes. Eastern Central Asia is far from oceans and highlighted as a typical temperate arid region. The aridity of this area is indicated to have originated in the Late Eocene, when the retreat of the Tethys began [24], and to have greatly intensified during the Quaternary [25]. Late Quaternary glacial-interglacial cycles also had strong effects in this region [18]. At the present, Eastern Central Asia is characterized by multiple landscapes, which includes mountains, valleys and desert basins. In high altitude mountains, such as the Tianshan and Altai ranges, climate is generally cold and humid, and coniferous forests cover the mountains and higher elevation valleys. By contrast, low altitude foothills and basins are strongly warm and dry; steppe and desert-steppe vegetation is distributed in these arid habitats.

To investigate the effect of climate change on plant species in arid Eastern Central Asia, we sampled two related species representing these contrasting vegetation types. *Clematis sibirica* is a woody perennial vine that grows primarily under conifer forests [26]; it has thin papery, denticulate 2-ternate leaves. This species is widespread in the Tianshan, Altai, and Western Dzungarian mountains, at altitudes between 1,000 and 2,300 m a.s.l. *C. songorica* is a small perennial shrub occurring in the steppe and on gravelly slopes [26]. It develops subleathery, undivided leaves. This species is widespread in the foothills of the high mountains surrounding the Dzungarian Basin, and at the western border of the Tarim Basin. Its habitats cover an altitude range of 400–2,100 m a.s.l.

Examining the evolutionary history and demographic dynamics of these two species of *Clematis* during past climatic fluctuations can shed light on the way in which they may respond to climate change in the future [4], [27], [28]. Recently, molecular genetic approaches to the inference of evolutionary history and demographic dynamics have become available and useful for plant species lacking fossil records [29]. In Eastern Central Asia, alpine glaciers have extensively advanced to piedmont of mountains during the Quaternary glacial periods [18], [30]. But it is different from the ice sheets in northern Europe and North America. Previous studies indicate that these paleoclimatic fluctuations have affected plant distribution and evolution in Eastern Central Asia both from the fossil record and molecular evidence [21], [31]. Here, we assess the spatial genetic pattern of each of the study species using both a biparentally inherited nuclear marker and a maternally inherited cpDNA marker [32]. In the angiosperm species, the nuclear genome is inherited from both pollen and seed, while the chloroplast genome is only inherited from seed. The different modes of inheritance have resulted in higher level of gene flow in the nuclear genome than that in the chloroplast genome. In addition, species distribution modelling (SDM), interpreting the distribution of species relying on multiple layers of climatic data, is an appropriate approach for examining a species’ range shift in the face of past and future climate changes [3], [33], although some projections could be overestimated [34]. Thus, in the present study, using *C. sibirica* and *C. songorica* as proxies of forest species and steppe species in arid Eastern Central Asia, we employ approaches of molecular phylogeography and SDMs to address the following questions:

1. What were the genetic and evolutionary consequences of glacial-interglacial cycles for *C. sibirica* and *C. songorica*? To what extent have ranges of the two species been reduced during the Pleistocene glaciations?
2. In the face of ongoing climate change, will these two plant species undergo loss of genetic diversity and range contraction?

## Materials and Methods

### Ethics Statements

No specific permits were required for this study. These study species did not involve endangered or protected species. The localities, where the populations were collected, are no private or protected areas.

### Species study and population sampling

**Figure 1:**
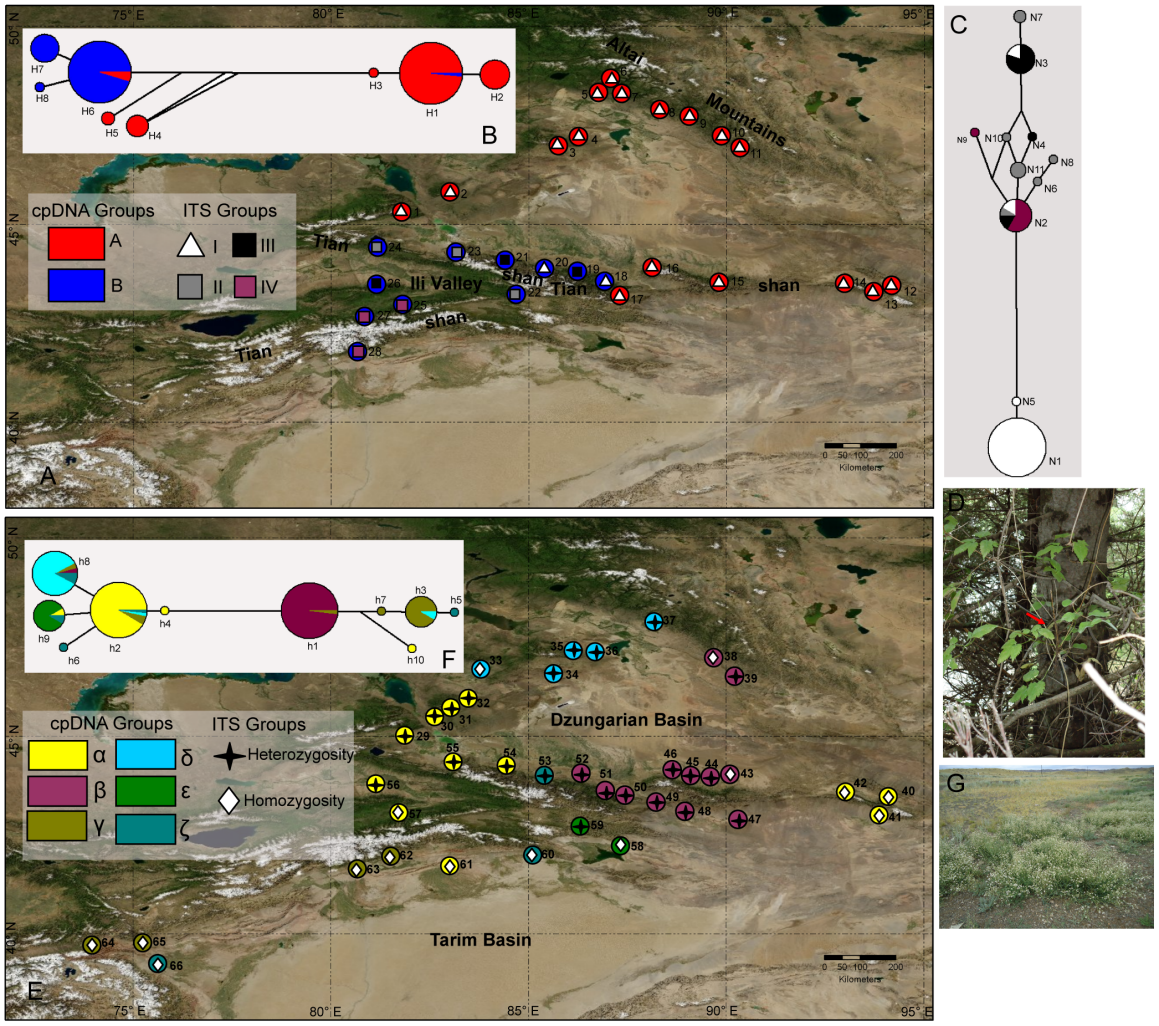
Sampling locations and distributions of population groups in *Clematis sibirica* (A) and *C. songorica* (E) in arid Eastern Central Asia. Population codes are consistent with the population names shown in Table S1. The color of each pie corresponds to the cpDNA population groups; the symbols with different colors correspond to the nrITS population groups. (B, F) Median-joining networks for recovered chlorotypes in these two species. The colors within each chlorotype are consistent with the cpDNA population groups. (C) Median-joining networks for recovered ribotypes in *Clematis sibirica*. The colors within each ribotype are consistent with the nrITS population groups. (D, G) Habitats of these two species.

The two related *Clematis* species, *C. sibirica* (Figure 1A) and *C. songorica*, were examined in this study. The former favors humid and shady forest habitats, while the latter can adapt to strongly arid, high-light conditions. We sampled 28 populations of *C. sibirica* and 38 populations of *C. songorica,* with 1 to 5 individuals per population (Table S1; Figure 1). The *C. sibirica* populations were collected in Zhang and Zhang [35]. This intensive sampling covered most of the study areas. Fresh leaf material was dried immediately in the field using silica gel.

### DNA extraction, amplification and sequencing

We extracted total genomic DNA from approximately 50 mg of silica-dried sample leaves following a CTAB protocol modified from Doyle and Doyle [36]. The cpDNA fragment *psb*A-*trn*H [37] was amplified for the individuals of *C. songorica*; sequences of *psb*A-*trn*H for *C. sibirica* were available from a previous study [35]. The nuclear ITS region [38] was amplified for the individuals of both species. The PCR mixture and amplification program followed the protocols of Zhang and Zhang [21]. PCR products were purified with Purification Kits (Qiagen), following the recommended protocol. Sequencing reactions were conducted on an ABI 3730 automated sequencer in Sangon Biotech Co., Ltd., Shanghai, China. Based on the chromatograms of both reading directions of sequences, we had detected three additive nucleotides and identified three heterozygous genotypes for nrITS sequences in 43 of the total 116 individuals of *C. songorica*. Such infra-genomic polymorphisms in nuclear DNA (nDNA) have commonly been used to identify lineage hybridizations [39], [40]. Here, we treated these individuals with peak additivity patterns in the chromatograms as heterozygotes. CLUSTAL_X [41] was used to align the generated sequences, and then they were checked manually. Newly identified sequences were deposited in the GenBank Database under accession numbers KC415696–KC415727.

### Population genetic structure and demographic analyses

To understand levels of genetic diversity in *C. sibirica* and *C. songorica*, we have calculated two indices, chlorotype/ribotype diversity (h) and nucleotide diversity (π), using Arlequin version 3.11 [42]. Due to the heterozygosity of nrITS in *C. songorica*, we have computed its unbiased expected heterozygosity (H_e_) based on the data matrices of alleles using GenAlEx 6.5 [43]. We have assessed genetic differentiation and tested presence of phylogeographical signal across the whole ranges of these two species by calculating *F*_ST_ (Wright’s fixation index) and comparing this index with *N*_ST_ for both cpDNA and nrITS datasets. *F*_ST_ is a measure of genetic differentiation based on chlorotype/ribotype frequencies; analogously, *N*_ST_ measures genetic differentiation based on the genetic distance between genotypes. A phylogeographical structure is present when *N*_ST_ is larger than *F*_ST_ [44]. Allele distance matrix was computed in PAUP* v.4.0b10 [45] with the character of mean differences among alleles. The values of these two indexes and their significances were computed and tested by constructing the distribution of the null hypothesis by means of 999 random permutations of individuals among populations using SPAGeDi 1.3 [46]. The presence of a phylogeographical signal was tested by assessing the significance of the observed difference between *N*_ST_ and *F*_ST_ values by means of 999 random permutations of the allele distance matrix.

We further compared the patterns of genetic differentiation between cpDNA loci and nrITS at the scale of individuals across the sampling ranges of these two species along gradients of geographical distance in order to infer different role and importance of seed- and pollen- mediated gene flow in the forest and steppe species during their post-glacial histories. For that purpose, we calculated pairwise kinship coefficients among individuals, *F*_ij_, using J. Nason’s estimator [47], as well as *N*_ij_, which is analogous to *F*_ij_ and considers the phylogenetic relationship among alleles. *F*_ij_ and *N*_ij_ were computed from allele frequencies across the sampling ranges of these two species. To test the significance of isolation by distance (IBD), slope of the regression of *F*_ij_ or *N*_ij_ on the logarithm of spatial distance among individuals, ln(*d*_ij_), was assessed by means of 999 random permutations of population locations (Mantel test). Threshold distance was divided into several intervals: for *C. sibirica*, they were 0, 100, 250, 500, 750 and 1000 km; for *C. songorica*, they were 0, 100, 250, 500, 750, 1000 and 1500 km. The mean *F*_ij_ or *N*_ij_ values were also computed over i, j pairs separated by predefined geographical distance intervals, d, giving *F*_(d)_ and *N*_(d)_. The difference between *N*_(d)_ and *F*_(d)_ was tested by means of 999 random permutations of the allele distance matrix in order to determine the presence of a phylogeographical signal at different spatial scales. All analyses were performed in SPAGeDi 1.3 [46].

We conducted two types of analyses for chlorotypes/ribotypes in *C. sibirica* and chlorotypes in *C. songorica* as implemented in Arlequin version 3.11 [42] to examine possible demographic expansions within each of the species. In the first method, Tajima’s *D* [48] and Fu’s *F*_S_ [49] were calculated to assess possible expansions; large negative values of *D* and *F*_S_ statistics are in accordance with the expansion hypothesis. Significance was calculated using 10,000 replicates. In the other method, pairwise mismatch distributions [50] were calculated to examine the demographic expansions of the two plant species. We used parametric bootstrapping with 1,000 replicates and tested for goodness-of-fit against a model of sudden expansion [42], to calculate the sum of squared deviations (SSD) and raggedness index (HRag) [51] between observed and expected mismatch distributions and to obtain τ with 95% confidence intervals (CIs).

Genetic landscape shapes analysis was also performed to investigate patterns of change of genetic differentiation with geographical distance. This analysis was conducted using Alleles In Space software [52]. The procedure initially constructs a Delaunay triangulation network among all the sampled populations, after which the average inter-individual genetic distances were calculated between populations in the network. Next, a simple interpolation procedure was used to infer genetic distances at locations on a uniformly spaced grid covering the entire sampled landscape. A three-dimensional surface plot (X and Y coordinates correspond to geographical locations and Z to genetic distances) of the set of interpolated genetic distances was generated.

We constructed median-joining networks using the program Network version 4.6 [53] to detect genealogical relationships among the identified chlorotypes/ribotypes of *C. sibirica* and chlorotypes of *C. songorica*. In this analysis, each indel was treated as a single mutation event. To define geographical structure of sampled populations, a spatial analysis of molecular variance was carried out in SAMOVA 1.0 [54] for chlorotypes/ribotypes of *C. sibirica* and chlorotypes of *C. songorica*. This program implements a simulated annealing approach to gather geographically homogenous populations and maximally differentiate them from each other within defined groups of populations (*K*). The simulated annealing process was run for 10,000 iterations for 2 ≤ *K* ≤ 20, from 200 initial conditions. An *F*_CT_ value was given for every calculation. We identified the structure of population groups that did not include any groups of a single population, when the maximum value of *F*_CT_ value was given [55], [56]. If one or more single-population groups were included, it indicates that the group structure was disappearing [55], [56].

### Molecular dating approach and lineage through time analyses

The genus *Anemone* was chosen as outgroup in the phylogenetic analyses for both cpDNA and nrITS, based on previous molecular [57] and morphological [58] studies on Ranunculeae. The sequences (*psb*A-*trn*H and ITS) of *Anemone* were taken from the GenBank Database. A likelihood-ratio test in MEGA 5.05 [59] was employed to examine whether the four sets of chlorotype/ribotype sequences fitted the hypothesis of a molecular clock, by comparing the log likelihood of the ML trees (HKY, uniform rates) with and without molecular clock constraints. In this test, a molecular clock was not rejected at a 5% significance level for the three datasets: the chlorotype/ribotype sequences of *C.sibirica* (cpDNA: Δ*ln*L  =  4.8, *P*<0.21; nrITS: Δ*ln*L  = 8.3, *P*<0.08), and the chlorotype sequences only of *C. songorica* (Δ*ln*L  = 3.9, *P*<0.54). We then used the program BEAST version 1.6.1 [60] to date genetic divergence. BEAST employs a Bayesian Markov chain Monte Carlo (MCMC) approach with a fixed molecular clock, a coalescent tree prior, and a HKY substitution model for these three datasets. The MCMC chains were run for 10,000,000 generations, sampling every 1,000 generations. The combined parameters were checked in Tracer version 1.4 [60]. Effective sample sizes (ESS) for the relevant estimated parameters were well above 200. Finally, trees were edited in FigTree v.1.3.1 (http://tree.bio.ed.as.uk/software/figtree/). The cpDNA substitution rates for most angiosperm species have been estimated to vary between 1 and 3×10^–2^ substitutions per site per million years [61]. For the nrITS region, substitution rates in herbaceous plants have been estimated to vary from 3.46×10^–2^ to 10×10^–2^ substitutions per site per million years [62]. Based on the uncertainties of these rate values, we used normal distribution priors and set the parameters as a mean of 2×10^–2^ and a SD of 6.08×10^–3^ for cpDNA, and a mean of 6.73×10^–2^ and a SD of 1.99×10^–2^ for ITS to cover these rate ranges within the 95% range of the distribution to estimate the divergence times of chlorotypes/ribotypes.

To obtain estimations of historical demographic changes through time, Extended Bayesian Skyline Plot (EBSP) analysis, as implemented in BEAST version 1.6.1 [60], was employed with all individuals in three datasets: cpDNA and nrITS sequences of *C. sibirica,* and cpDNA sequences of *C. songorica*. This coalescent-based approach uses Markov chain Monte Carlo (MCMC) sampling procedures to estimate the posterior distribution of effective population size during a time period [60]. We used the same substitution rates of *psb*A-*trn*H (cpDNA fragment) and the nrITS region as in the foregoing dating approach. MCMC analyses were run for 50,000,000 generations, sampling every 1,000 generations and discarding 1,000 samples as burn-in. We generated the lineage through time (LTT) plots of each dataset in Tracer version 1.4 [60].

### Species distribution modelling

To obtain the historical, current and future distribution ranges of *C. sibirica* and *C. songorica*, the species distribution modelling (SDM) approach was carried out to determine their potential distributions at the Last Glacial Maximum (LGM; c. 21 ka before present), the present-day, and the future (during the 2080’s) using the maximum entropy approach as implemented in the program MAXENT 3.3.3k [63].

Present-day species occurrence data with geographical coordinates for these two plant species were available from our field surveys and the Chinese Virtual Herbarium (CVH; http://www.cvh.org.cn/cms/). In total, 49 points of *C. sibirica* and 78 points of *C. songorica* were used in these modelling analyses. We obtained current bioclimatic layers with 19 bioclimatic variables at a resolution of 2.5 arcmin from the WordlClim database (available at http://www.worldclim.org/download). When species distribution modelling is employed, strong colinearity of environmental variables could result in model over-fitting [64]. Therefore, we examined the Pearson correlation for bioclimatic variables. To avoid this defect, the least correlated variables (Pearson correlation value <0.8) were retained. At last, 9 remaining bioclimatic variables were used to model the species distributions. To find the optimal input parameters in Maxent for these data sets, we ran models with different options for regularization multiplayer, percentage of test sample and number of replicates. We identified the best model based on the area under the receiver operating characteristic curve (AUC) statistic value. The input parameters were chosen as regularization multiplayer  =  1, percentage of test sample  =  25%, number of replicates  =  15. A jackknife test was performed to measure the percent contributions of bioclimatic variables on the prediction for the distributional models. To obtain their LGM and future potential distributions, corresponding climatic datasets were used in the species distribution modelling approach. The data set for LGM climate was downloaded from the Paleoclimate Modelling Intercomparison Project Phase II [65]. The LGM bioclimatic layers based on the Model for Interdisciplinary Research on Climate (MIROC) [66] and the Community Climate System Model (CCSM) [67] were used to construct the LGM distribution of these two plant species. Future climatic scenarios during the 2080’s were derived from climate model outputs according to three scenarios (A1, A2, and B2) proposed by the Intergovernmental Panel on Climate Change Data Distribution Centre [68]. This distribution modelling was projected onto climate reconstructions for both the LGM and a future time, with the same resolution of bioclimatic variables. The predicted presence of each species under each climatic scenario was defined in Maxent using a threshold approach. We used the fixed “10 percentile presence’’ threshold during the SDMs approaches because presence-only data were available here. In order to estimate range changes for each time period relative to the present condition as a percentage of occupied area, cells with predicted occurrences were counted for each projection in ArcMap 10 (ESRI, California, USA).

## Results

### Population genetic structure and demographic analyses

Based on nucleotide variations of cpDNA and nrITS sequences, we distinguished chlorotypes and ribotypes in the 28 populations of *C. sibirica* and chlorotypes in the 38 populations of *C. songorica*. In total, eight chlorotypes and eleven ribotypes were identified in *C. sibirica*, while ten chlorotypes were identified in *C. songorica* (Table 1; Table S2). For nrITS dataset of *C. songorica*, eight alleles and three heterozygote genotypes were detected (Table S2). Higher levels of chlorotype diversity and chloroplast nucleotide diversity were shown in *C. songorica* than *C. sibirica* (0.7407 vs. 0.6712 and 0.024547 vs. 0.016635; Table 1). With respect to nucleotide diversity of nrITS datasets, a higher level of diversity was shown in *C. sibirica* than in *C. songorica* (0.013148 vs. 0.001151; Table 1). The level of ribotype diversity in *C. sibirica* was also higher than unbiased expected heterozygosity in *C. songorica* (0.4963 vs. 0.3612; Table 1). In *C. sibirica*, a significant phylogeographical structure and high level of genetic differentiation across the whole range of this species were supported based on both cpDNA (*F*_ST_ =  0.4304, *P*<0.001; *N*_ST_ > *F*_ST_, *P*<0.05; Table 1) and nrITS (*F*_ST_ =  0.6265, *P*<0.001; *N*_ST_ > *F*_ST_, *P*<0.05; Table 1). By comparison, the level of genetic differentiation across the whole range of *C. songorica* was high for cpDNA (*F*_ST_ =  0.8250, *P*<0.001; Table 1), but low for nrITS (*F*_ST_ =  0.2778, *P*<0.001; Table 1). A significant phylogeographical pattern of *C. songorica* was rejected by both of these two datasets (Table 1).

**Table 1:** Estimates of chlorotype/ribotype diversity (h), unbiased expected heterozygosity (H_e_), nucleotide diversity (π) and two genetic differentiation indexes (*F*_ST_, *N*_ST_) in *Clematis sibirica* and *Clematis songorica*.

		cpDNA						nrITS					
Species	*N*_pop_	*N*_ind_	n	h (±SD)	π (±SD)	*F*_ST_	*N*_ST_	*N*_ind_	n/A	h/H_e_ (±SD)	π (±SD)	*F*_ST_	*N*_ST_
*Clematis sibirica*	28	125	8	0.6712 (0.0282)	0.016635 (0.00894)	0.4304	0.6581 (*P* = 0.046*)	109	11	0.4963 (0.0552)	0.013148 (0.00689)	0.6265	0.7866 (*P* = 0.007*)
*Clematis songorica*	38	169	10	0.7407 (0.0180)	0.024547 (0.01265)	0.8250	0.7922 (*P* = 0.478)	116	8	0.3612 (0.0458)	0.001151 (0.00102)	0.2778	0.2094 (*P* = 0.926)

* indicates significance at less than 0.05 *P*-value.

The significance test associated with *N*_ST_ results from the randomization of the genetic distance matrix among alleles and measures whether *N*_ST_ > *F*_ST_. Numbers of populations (*N*_pop_), sequenced individuals (*N*_ind_) and chlorotypes/ribotypes (n) or alleles (A) in heterozygotes were shown.

Average kinship coefficients between pairs of individuals showed a decrease trend along with a distance gradient for both the cpDNA and nrITS in *C. sibirica* (Figure S1). In *C. songorica*, average kinship coefficients were decreased along with a distance gradient for cpDNA, but increased for nrITS (Figure S1; Table 2). IBD analyses indicated that an isolation by distance pattern for cpDNA/nrITS presented in *C. sibirica* at less than 500/250 km scale (Table 2). With respect to *C. songorica*, the isolation by distance pattern for cpDNA/nrITS was presented at less than 250 km or more than 1000 km scale (Table 2).

**Table 2:** Slope (and *P*-value) of the regression analyses between pairwise *N*_ij_ values in relation to a geographical distance gradient and phylogeographical signal (*N*_ij_ > *F*_ij_ test) at different spatial classes for cpDNA and nrITS datasets in *Clematis sibirica* and *Clematis songorica*.

	cpDNA		nrITS	
Species	Slope	*P*-value of *N*_ij_ > *F*_ij_	Slope	*P*-value of *N*_ij_ > *F*_ij_
*Clematis sibirica*	−1.121 10^−3^ (0.084)	0.007 for distances <500 km	−1.191 10^−3^ (0.078)	0.006 for distances <250 km
*Clematis songorica*	−6.071 10^−4^ (0.256)	<0.001 for distances <250 km	2.729 10^−4^ (0.030)	0.014 for distances >1000 km

**Figure 2:**
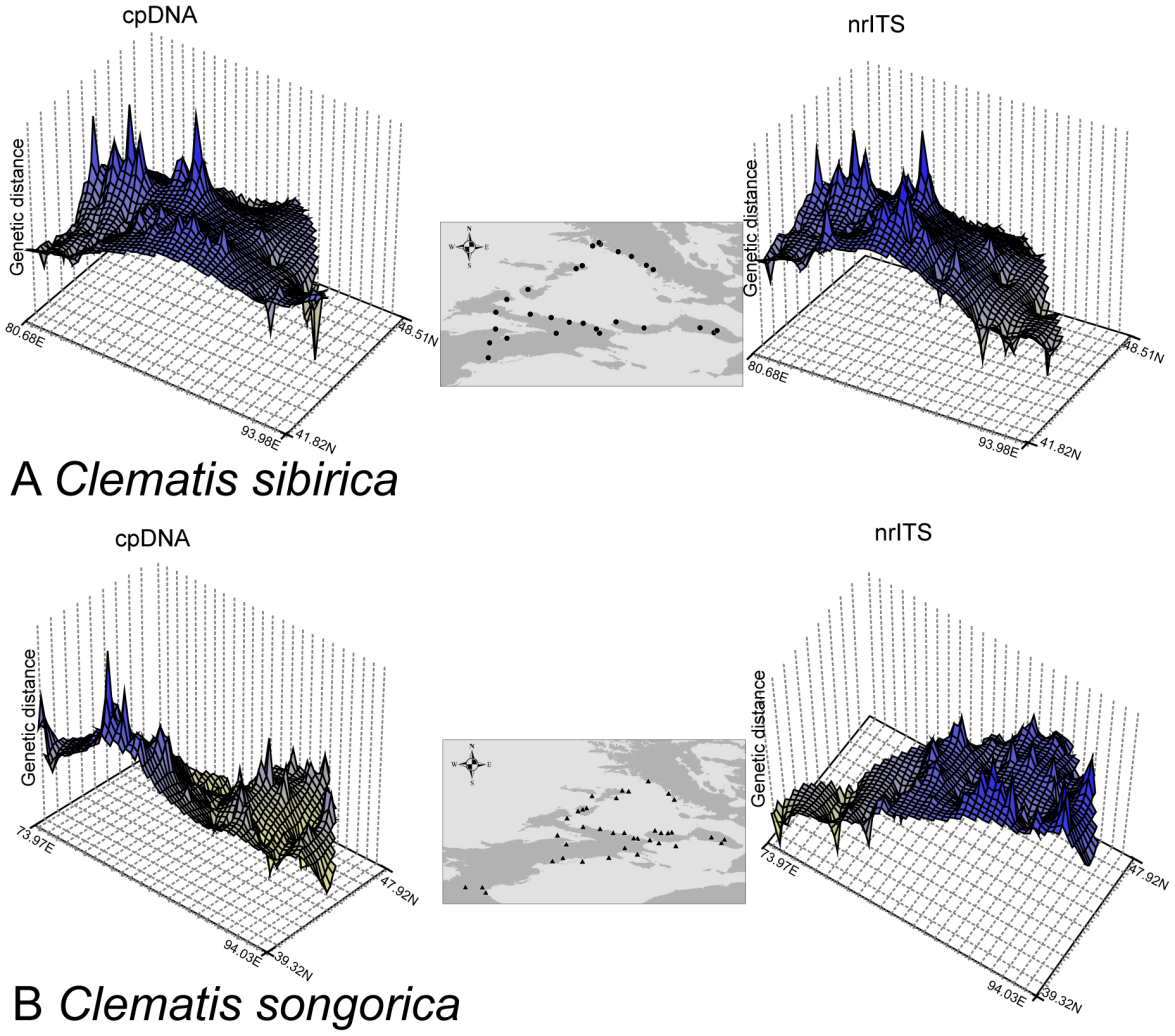
Spatial genetic landscapes constructed from the cpDNA sequences (left) and nrITS (right) sequences across the total distributions of *Clematis sibirica* (A) and *C. songorica* (B).

The surface plots of cpDNA and nrITS sequence data for *C. sibirica* produced by genetic landscape shape interpolation analyses showed lower levels of genetic differentiation among eastern populations than that among western populations (Figure 2A). By contrast, *C. songorica* displayed consistent differentiation among all of the sampled populations, in the surface plots of both the cpDNA and nrITS datasets (Figure 2B). At the species level, a recent expansion model was rejected for *C. sibirica* and *C. songorica* based on Tajima’s *D* and Fu’s *F*_S_ values (Table 3), and neutrality tests and bimodal mismatch distributions (not shown) for cpDNA and nrITS sequences.

**Table 3:** The results of Tajima’s *D* and Fu’s *F*_S_ tests, and mismatch distribution analyses for chlorotypes/ribotypes of *Clematis sibirica* and chlorotypes of *Clematis songorica*.

	Tajima’s *D* test		Fu’s *F*_S_ test		Mismatch distribution					
	*D*	*P*	*F*_S_	*P*	τ	95% CI	*Hrag*	*P*	SSD	*P*
*Clematis sibirica*										
Chlorotypes	0.905	0.840	7.436	0.965	11.281	0–64.281	0.204	0.019	0.151	0.059
Ribotypes	2.117	0.971	6.106	0.936	0	0–0.566	0.391	0.939	0.347	< 0.001
*Clematis songorica*										
Chlorotypes	1.179	0.886	12.130	0.977	14.201	0.299–26.225	0.158	0.006	0.125	0.042

### Population partitions and molecular divergence time estimation

**Figure 3:**
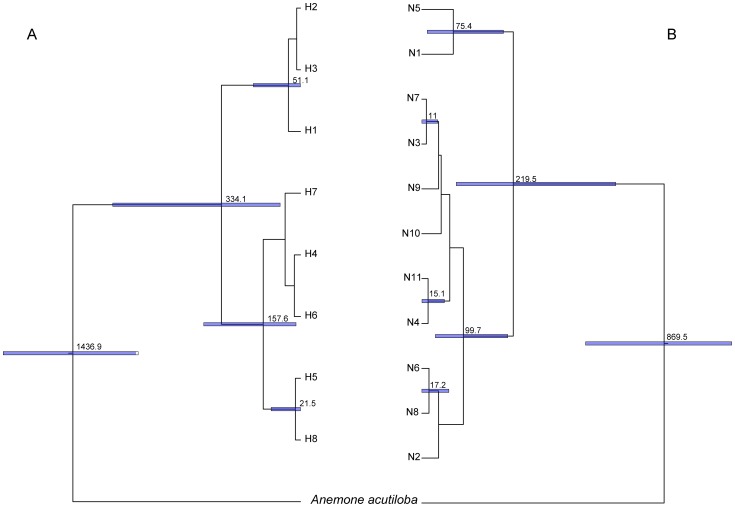
Divergence time (ka BP.) of *Clematis sibirica* in cpDNA (A) and nrITS (B) datasets based on BEAST analysis.

Based on the spatial distribution of eight chlorotypes in *C. sibirica*, these 28 sampled populations were divided into two geographical groups: Group A and Group B (Figure 1A). Populations of Group A are distributed in the Western Dzungarian mountains, the Altai Mountains and the eastern Tianshan Mountains. Populations of Group B are located in the western part of the Tianshan Mountains. The median-joining network showed a genetic barrier with infrequently shared chlorotypes between these two population groups (Figure 1B). Molecular dating analysis estimated that these chloroplast genetic divergences in *C. sibirica* occurred in the range from 21.5 ka to 334.1 ka (Figure 3A). Evidence from the distribution of ribotypes in *C. sibirica*, four geographical groups (Group I to Group IV) emerged in these 28 sampled populations (Figure 1A). Populations of Group I are mostly equal to these of chloroplast Group A. The median-joining network of eleven ribotypes exhibited the genetic divergence between Group I and other three groups (Figure 1C). Divergence time of these eleven ribotypes was estimated in the range from 11 ka to 218.5 ka (Figure 3B).

The 38 populations of *C. songorica* were clustered into six groups (Group α to Group ζ; Figure 1E) based on cpDNA data. These six groups aren’t well consistent with geographic regions. In the median-joining network of chlorotypes (Figure 1F), each of these six population groups mostly occurred in a haplotype node. Molecular dating analysis showed these genetic divergences occurred in the time range from 28.5 ka to 462.8 ka (Figure S2). For the nrITS dataset of *C. songorica*, heterozygotes existed in 23 of these 38 sampled populations (Figure 1E). Lineage-through-time (LTT) plots of cpDNA and nrITS sequences for *C. sibirica* are shown in Figure S3. They indicate that rapid lineage accumulation for this species occurred after ca. 50 ka BP. The LTT plots of cpDNA sequences for *C. songorica* indicate that its rapid lineage accumulation began at ca. 100 ka BP (Figure S3).

### Past, current and future species distributions

The selected model for *C. sibirica* had an average AUC value of 0.994±0.002 over the 15 replicate runs. The bioclimatic variables that contributed most to the distribution modelling of *C. sibirica* were: Temperature Annual Range (22.8%), Mean Temperature of Driest Quarter (22.5%), Mean Temperature of Coldest Quarter (15.9%), Precipitation of Driest Quarter (15.7%) and Isothermality (13.3%). For the selected model of *C. songorica*, a mean AUC value of 0.993±0.002 was obtained over the 15 replicate runs. The bioclimatic variables with highest relative contribution to the SDMs for *C. songorica* were Annual Mean Temperature (19.1%), Precipitation of Wettest Quarter (18.9%), Precipitation of Driest Quarter (17.4%), Mean Temperature of Driest Quarter (16.1%) and Mean Temperature of Coldest Quarter (10.9%).

**Figure 4:**
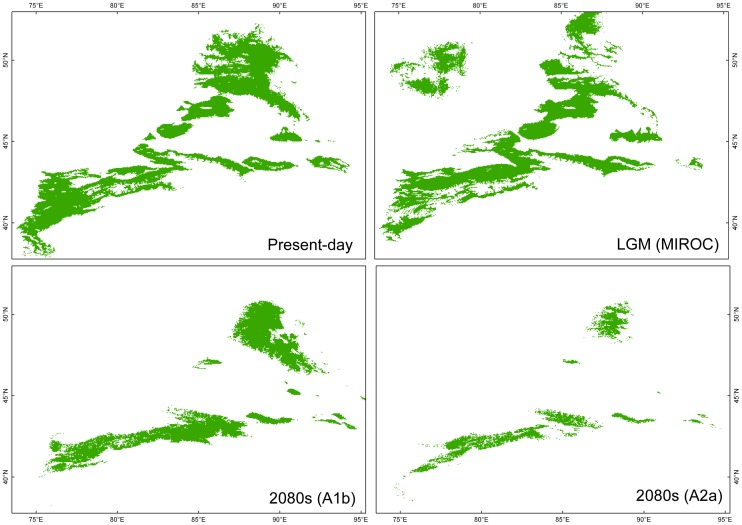
Modelling distribution of *Clematis sibirica*. Maps depicting potential distribution of *Clematis sibirica* in arid Eastern Central Asia during present-day and at LGM based on the MIROC model, and future (2080’s) based on the A1b and A2a scenarios.

**Figure 5:**
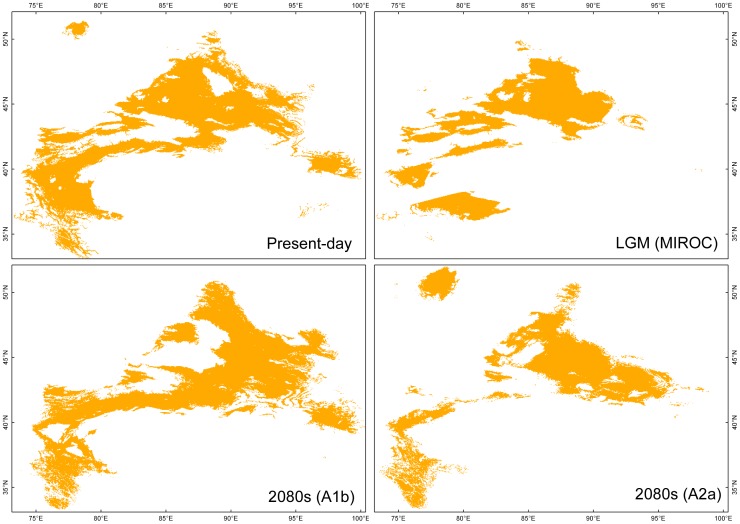
Modelling distribution of *Clematis songorica*. Maps depicting potential distribution of *Clematis songorica* in arid Eastern Central Asia during present-day and at LGM based on the MIROC model, and future (2080’s) based on the A1b and A2a scenarios.

The present potential distribution of *C. sibirica* appeared in the Altai Mountains, the Tianshan Mountains and the Western Dzungarian mountains (Figure 4). For *C. songorica*, its present potential distribution had a large area including the Dzungarian Basin and the western border of the Tarim Basin (Figure 5). LGM projections of these two species did not give credible results based on the CCSM model (not shown), which showed small distributions for these two species. In comparison with present potential distribution, MIROC projection of *C. sibirica* indicated it did not undergo extensive range changes during the LGM period (Table 4; Figure 4). Although the LGM projected distribution of *C. songorica* was reduced (about 60% of its present potential range; Table 4), this species did not experience intensive retreating or migration (Figure 5). The models of these two species for 2080’s based on the A2 and B2 climatic scenarios yielded less range areas than that based on the A1 climatic scenario. For *C. sibirica*, the predicted distributions indicated a large-scale reduction of suitable distribution areas during the 2080’s in comparison with its present potential distribution (Figure 4 and Table 4). In contrast, the SDMs of *C. songorica* showed a little shift between the present potential distribution and the predicted distributions during the 2080’s (Figure 5 and Table 4).

**Table 4:** Percentage of area maintained as compared to the present potential distribution area in the *Clematis sibirica* and *Clematis songorica* according to past (LGM using the MIROC projection) and future (2080s; A1b, A2a and B2a scenarios) modelled distribution ranges.

	*Clematis sibirica*	*Clematis songorica*
LGM (MIROC) vs. Present	98.97%	60.00%
2080s (A1b) vs. Present	69.91%	115.51%
2080s (A2a) vs. Present	26.42%	69.19%
2080s (B2a) vs. Present	35.91%	70.55%

## Discussion

### Influence of past climatic changes on phylogeographical structures of *Clematis sibirica* and *C. songorica*

A significant phylogeographical signal (*N*_ST_ > *F*_ST_, *P*<0.05; Table 1) was present in *C. sibirica* based on the analyses of cpDNA and nrITS datasets. Our dating results suggested that genetic divergences within *C. sibirica* occurred during the mid- and the late Pleistocene (Figure 3), which is well consistent with the glaciation process in the study area [18]. From the genetic and dating evidence, we believe that Pleistocene climatic fluctuations had profoundly influenced the phylogeographical structure of *C. sibirica*. In respect to other species in the study area [20], [69], the Pleistocene climate changes have likely played an important role in shaping their spatial genetic structures and distribution patterns as well.

A decrease of cpDNA and nrITS kinship coefficients between pairs of individuals along a gradient of geographical distance (Figure S1) suggested that seed- and pollen- mediated gene flow contributed equally to the phylogeographical divergence of *C. sibirica*. These 28 populations of *C. sibirica* were grouped into two partitions (Figure 1A) using the cpDNA dataset: the eastern Tianshan Mountains & Altai Mountains (Group A) and the western Tianshan Mountains (Group B). The phylogenetic network of chlorotypes (Figure 1B) demonstrated a genetic barrier with few shared genotypes between these two groups. According to the nrITS dataset, four groups were divided for the 28 sampled populations (Figure 1A). Populations of nuclear Group I were mostly equal to these of chloroplast Group A, while other populations were divided into three related groups. Group I covered the eastern Tianshan Mountains and the Altai Mountains; other three groups located in the western Tianshan Mountains. In the phylogenetic network of ribotypes (Figure 1C), a genetic divergence was obvious between Group I and other three groups. Based on the population partitions and distribution of genotypes from cpDNA and nrITS datasets, we divided these 28 populations of *C. sibirica* into two phylogeographical groups: Group of the eastern Tianshan Mountains & Altai Mountains and Group of the western Tianshan Mountains. During glacial periods, the climatic processes of cooling and aridity occurred simultaneously in Eastern Central Asia [18], [31]. In order to overcome the cold-dry glacial climate, the two phylogeographical groups of the forest species *C. sibirica* retreated to more mesic refugial areas [35]. The genetic landscape shape interpolation analyses of both cpDNA and nrITS sequences showed high levels of genetic differentiation among western populations (Figure 2A); the locations of the western populations are more humid than those of eastern populations in the study area [70]. Based on evidence from genetic diversity and humid environment, these western locations provided stable habitats, preserving the species’ existing genetic variation and maintaining genetic differences among populations. Populations of 1, 3, 5, 6 and 7 were presumed to be the refugial locations for Group of the eastern Tianshan Mountains & Altai Mountains [35]; analogously, populations of 22, 24, 25, 27 and 28 had provided refugia for Group of the western Tianshan Mountains [35]. In this area of arid Eastern Central Asia, at least three glacial-interglacial cycles were significantly undergone [18], [31]. The largest extent of glaciations occurred at the first glacial period. However, in the later two ice ages, the extent of glaciations became smaller and smaller [18]. During the later glacial periods, populations of *C. sibirica* may not have experienced confinement to refugia. This was supported by species distribution modelling (SDM), which showed similar ranges of this species under the LGM and the present climatic conditions (Figure 4 and Table 4).

Lineage-through-time (LTT) plots inferred from both cpDNA and nrITS sequences (Figure S3) suggest that *C. sibirica* has experienced rapid lineage accumulation since ca. 50 ka BP. This is associated with demographic expansion during the interglacial period after large glaciation processes in the study area [31]. Although the neutrality tests and bimodal mismatch distributions did not agree with a recent expansion for the whole of this species (Table 3), both of two phylogeographical groups were tested to have experienced demographical expansions in our previous study [35]. With the onset of the interglacial wetting and warming, the two groups of *C. sibirica* have expanded their ranges from western refugia and occupied arid eastern locations along the mountain corridors. Due to the influence of founder effect and genetic drift during the course of migration, these eastern populations display lower levels of genetic differentiation. The smoothness of the genetic landscape shape interpolation plots in the eastern part (Figure 2A) also supports low levels of genetic distance among these populations.

Based on the cpDNA and nrITS datasets, the presence of phylogeographical signal was rejected in *C. songorica* (*N*_ST_ < *F*_ST_, *P*>0.05; Table 1). It indicated that past climatic changes didn’t promote the complete lineage divergence of this species. A decrease of cpDNA kinship coefficients in the regression analysis, together with an increasing trend in the nrITS regression analysis, showed limited seed-mediated gene flow but high gene flow by mean of pollen dispersal in *C. songorica* (Figure S1; Table 2). SAMOVA had partitioned 38 sampled populations into six groups (Figure 1E) using the assembly of chlorotypes, which were not highly geographically structured. High significant genetic differentiation index (*F*_ST_  =  0.8250) of *C. songorica* in cpDNA shows distinct genetic divergence between populations. Because of the low level of gene flow inherent in plant chloroplast transmission [32], genetic divergence could occur between populations in stable habitats after a suitably long evolutionary history. By contrast, genetic differentiation index (*F*_ST_  =  0.2778) in nrITS demonstrates a high level of gene flow between populations for nuclear genes [32], and indicates there were no significant driving forces to promote nuclear divergence within this species. High levels of genetic distance among each of the 38 sampled populations was detected in the interpolation surface plots for both cpDNA and nrITS sequences (Figure 2B). This feature of genetic landscape shape analysis also indicates that this steppe species has enjoyed stable habitats during its past evolutionary history. Stable habitats, such as refugial regions, were less affected by climatic changes, and then they provided more opportunity to preserve allelic richness [71] and increase genetic differences between populations [10]. Based on the LGM and present-day climatic conditions, SDMs showed range contraction for *C. songorica* during the LGM period (Figure 5 and Table 4). Although the distribution area of this species was changed, the main distribution pattern of this species was still stable during the glacial period. Populations didn’t experience long-distance migrations. Thus, the distribution of this species didn’t display geographical divergence.

### Different sensitivity of these two species to climate changes and their future predictions

To predict how species respond to future climate changes, we need to confirm their dispersal ability under the past climatic conditions. In this study, we want to achieve this purpose by means of genetic and distribution modelling approaches. With respect to genetic evidences, the dispersal modes of seed and pollen can influence their genetic structures and their dispersal ability. For these two species in *Clematis*, fruits with a feathery tail are facilitated to flight via anemochory [72]. The feature of flowers that floral nectaries and petals are absent makes these two species to be between entomophily and anemophily. These two related species have similar dispersal modes of seed and pollen, which can eliminate the possibility that different dispersal modes promoted their various genetic structures. As evidenced from the higher levels of chloroplast genetic diversity and genetic differentiation (*F*_ST_) in *C. songorica* than that of *C. sibirica* (Table 1), *C. songorica* appears to have enjoyed greater stability of habitat, and to have maintained more stable population dynamics [10]. For the biparentally inherited nrITS sequences [32], lower levels of nuclear genetic differentiation (*F*_ST_) (Table 1) and widespread heterozygosity (Figure 1E) would imply that no significant divergence events occurred among populations in *C. songorica*. Benefiting from this stable demographic history in response to past climate change, it is suggested that *C. songorica* can stand firm in the face of climate change. On the other hand, *C. sibirica*, a montane forest species, underwent significant genetic divergence and long-distance retreat during the glaciation process in the Pleistocene. We suppose that the forest species would be much sensitive to climate changes than the steppe species and two main causes are responsible for this phenomenon. One is a higher ability to endure aridity and warming in steppe species. The variations of temperature and humidity to be expected may be within the limits of ecological amplitude of such species. The other is the different shape of distribution patterns between the two types of habitats. In arid Eastern Central Asia, forest species are distributed along mountain ranges at high altitudes, in narrow strip or corridor patterns (Figure 4), while steppe species have wide distributions within and around arid basins, often in larger patches with curvilinear boundaries (Figure 5). Based on landscape ecology principles, the larger, denser patches are effective in maintaining internal stability and resisting external disturbance [73].

In Eastern Central Asia, increased warming and variability of precipitation are expected in the future [23]. To what extent range shifts and genetic losses in forest and steppe species will occur under the threat of climate change is an important and useful question. The survival of plant species in forests and steppes is closely related to grazing and farming, as well as wild land environments in the area. *C. sibirica* and *C. songorica* can be treated as proxies of forest and steppe species, and used to provide insight into the impacts of climate change on these two ecological types of organisms. In order to well determine the reliability of predicting distribution changes, we have examined the possible mismatch between potential distribution and actual distribution. With the help of anemochory to transmit their seeds and pollen [72], both species could have high level of dispersal ability; in the study area, mountain ranges are connected with each other and basins are linked (Figure 1), which provide habitat connectivity for long-distance dispersal of these two species; using the genetic data, these two species also shows a high level of dispersal ability (about 250–500 km; Table 2) in the isolation by distance analysis. Therefore, the actual distributions of these two species would be much close to their potential distributions here.

SDMs predicted substantial differences between present-day and future (the 2080’s) potential distribution ranges of the forest species *C. sibirica* across the study area (Figure 4), based on a niche model, with a loss of about 30% of the potential habitat during future climatic conditions under A1b scenario as compared to its extant potential distribution (Table 4). Under A2a and B2a scenarios, it shows an over estimation that more than 65% of the present potential habitat will be disappeared during future climatic changes. In combined results of future climate from different models, temperature is projected to increase by 3–5°C, and will be accompanied by a further increase of aridity around the 2080’s in Central Asia [23]. *C. sibirica* was projected to reduce their potential distribution range in the southwestern Tianshan Mountains, the Western Dzungarian Mountains and the eastern Tianshan Mountains (Figure 4). However, the area of this species’ range increases around the humid Ili Valley (Figure 4). The Ili Valley served as a glacial refuge for the phylogeographical group of the western Tianshan Mountains [35]. This would be an example where refugia can provide safe havens for a species to overcome future climate change [74]. At the same time, in the Altai Mountains, this species remains intensively distributed with little range contraction (Figure 4). Climate projection indicates a slight decrease in precipitation over most of the study area, but a very slight increase in the northern part of Central Asia in the future [23]. Thus, many appropriate habitats will be maintained in the Altai Mountains. Although the potential distribution ranges of these two phylogeographical groups in *C. sibirica* are threatened to suffer severe decreases, loss of genetic diversity is not expected to occur. Locations with high levels of genetic diversity, such as the Ili Valley and the Altai Mountains, are shown to be still available under future climate change. Comparing the potential future distribution with its extant potential range (Figure 5 and Table 4), *C. songorica* will not experience significant decrease of species’ range in the face of the ongoing climate change. However, the species will possibly lose habitats in some western locations, but simultaneously extend its range in the east. Apart from these impacts on ecosystems, future climate change will also result in retreat of alpine glaciers in arid Eastern Central Asia [75].

In conclusion, we show that *C. sibirica* and *C. songorica* display different spatial genetic structure and demographic history in response to past climate changes. SDMs predict *C. sibirica* will face the challenge of potential range contraction in the future more than *C. songorica*. This can therefore be considered as an example of the distinct impacts of climatic change on forest and steppe plant species in arid lands. This study supports the hypothesis that an understanding of the evolutionary history and demographic dynamics of plant species during past climate change can shed light on hypotheses of how they will react in the face of ongoing change [4], [27], [28]. This study also provides perspective on ecological management in arid Eastern Central Asia, suggesting that increased attention should be paid to montane forest species due to their high sensitivity to disturbance.

## Supporting Information

Figure S1 **Mean** ***N*** **_ij_ (and associated significance test) along with a distance gradient for both the cpDNA and nrITS in** ***Clematis sibirica*** **(A) and** ***C. songorica*** **(B).**
(TIF)

Figure S2 **Divergence time (ka BP.) of** ***Clematis songorica*** **in cpDNA datasets based on BEAST analysis.**
(TIF)

Figure S3 **Lineage through time plots for** ***Clematis sibirica*** **based on cpDNA (A) and nrITS (B) datasets and** ***C. songorica*** **based on cpDNA (C) dataset.** Arrows indicate the occurrence of interglacial periods in the study area.
(TIF)

Table S1 **Details of sampling localities for the 28** ***Clematis sibirica*** **and 38** ***Clematis songorica*** **populations studied.** Population codes are numbered consecutively, as shown on the map in Figure 1. Number of individuals and genotype distribution in the cpDNA (cp*N*_ind_) and nrITS (nr*N*_ind_) analysis were shown.
(DOC)

Table S2 **Variable sites of each genotype for the cpDNA fragment (** ***psb*** **A-** ***trn*** **H) and the nrITS region in** ***Clematis sibirca*** **and** ***C. songorica*** **.**
(DOC)
