# Supplementary figures and images for: Retreating or Standing: Responses of Forest Species and Steppe Species to Climate Change in Arid Eastern Central Asia
Source: PLoS One. 2013 Apr 15;8(4):e61954. doi: 10.1371/journal.pone.0061954 (PMC3626637; doi:10.1371/journal.pone.0061954)

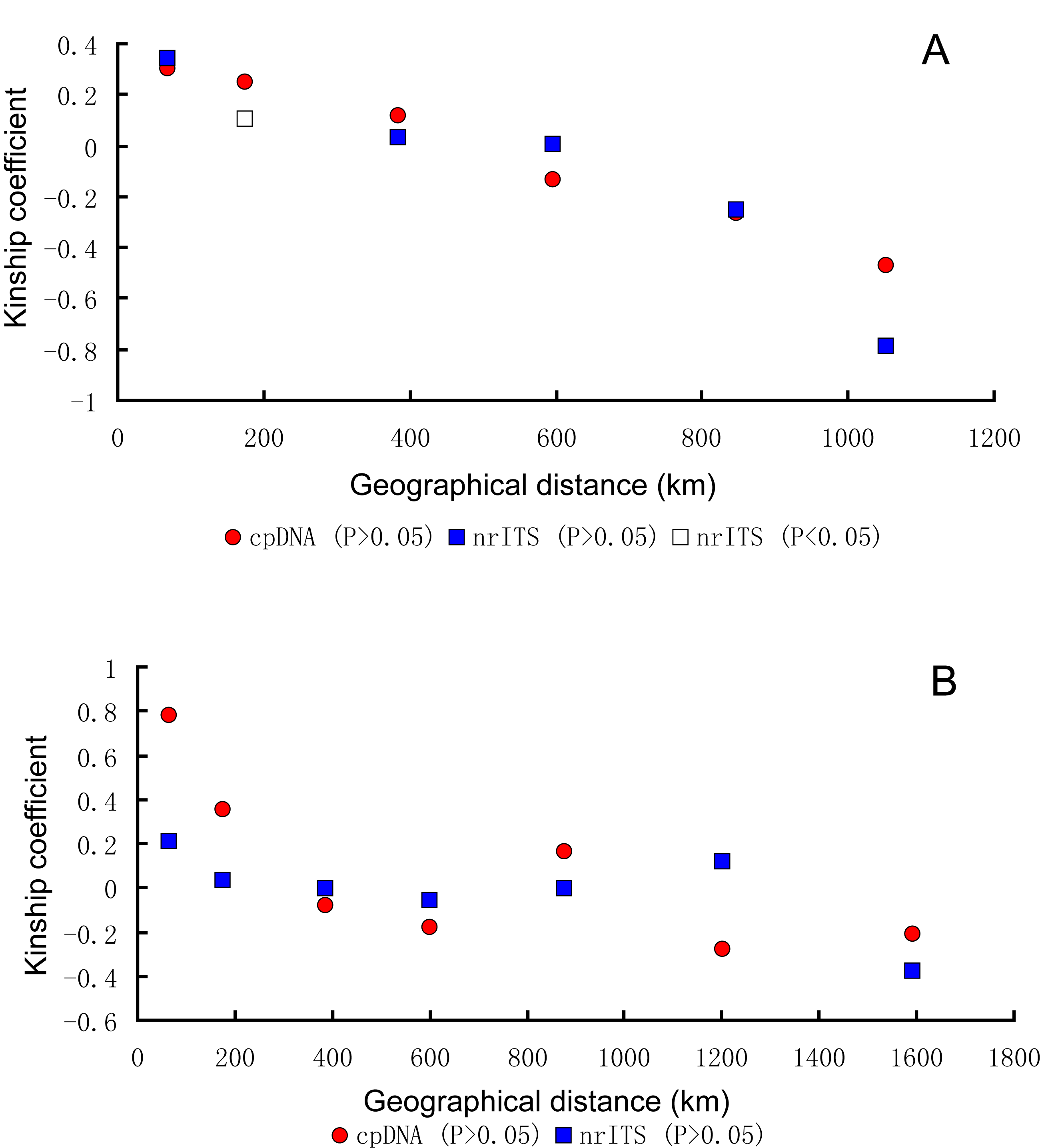

Supplement: Figure S1 — Mean N ij (and associated significance test) along with a distance gradient for both the cpDNA and nrITS in Clematis sibirica (A) and C. songorica (B). (TIF) [file pone.0061954.s001.tif]

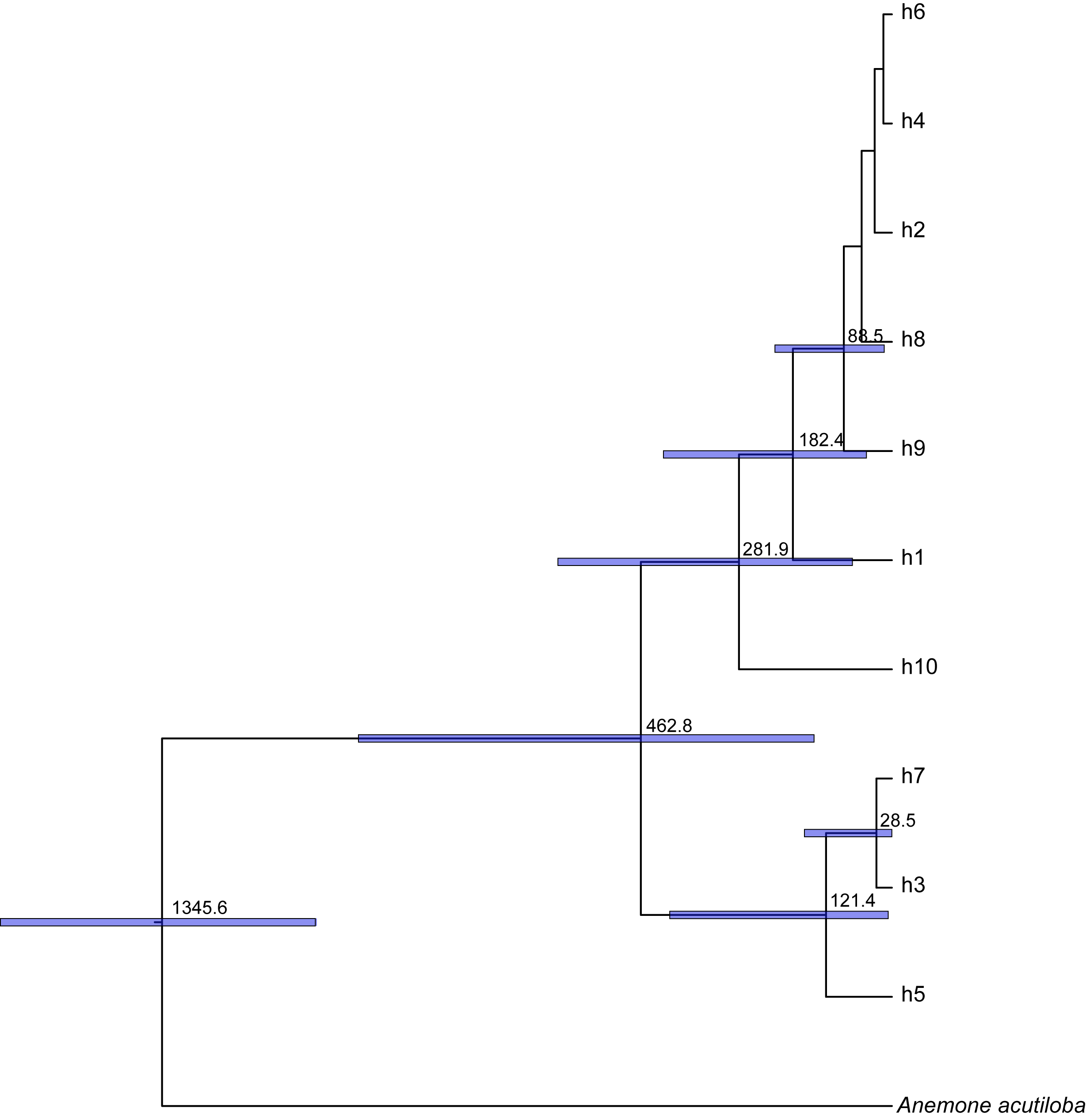

Supplement: Figure S2 — Divergence time (ka BP.) of Clematis songorica in cpDNA datasets based on BEAST analysis. (TIF) [file pone.0061954.s002.tif]

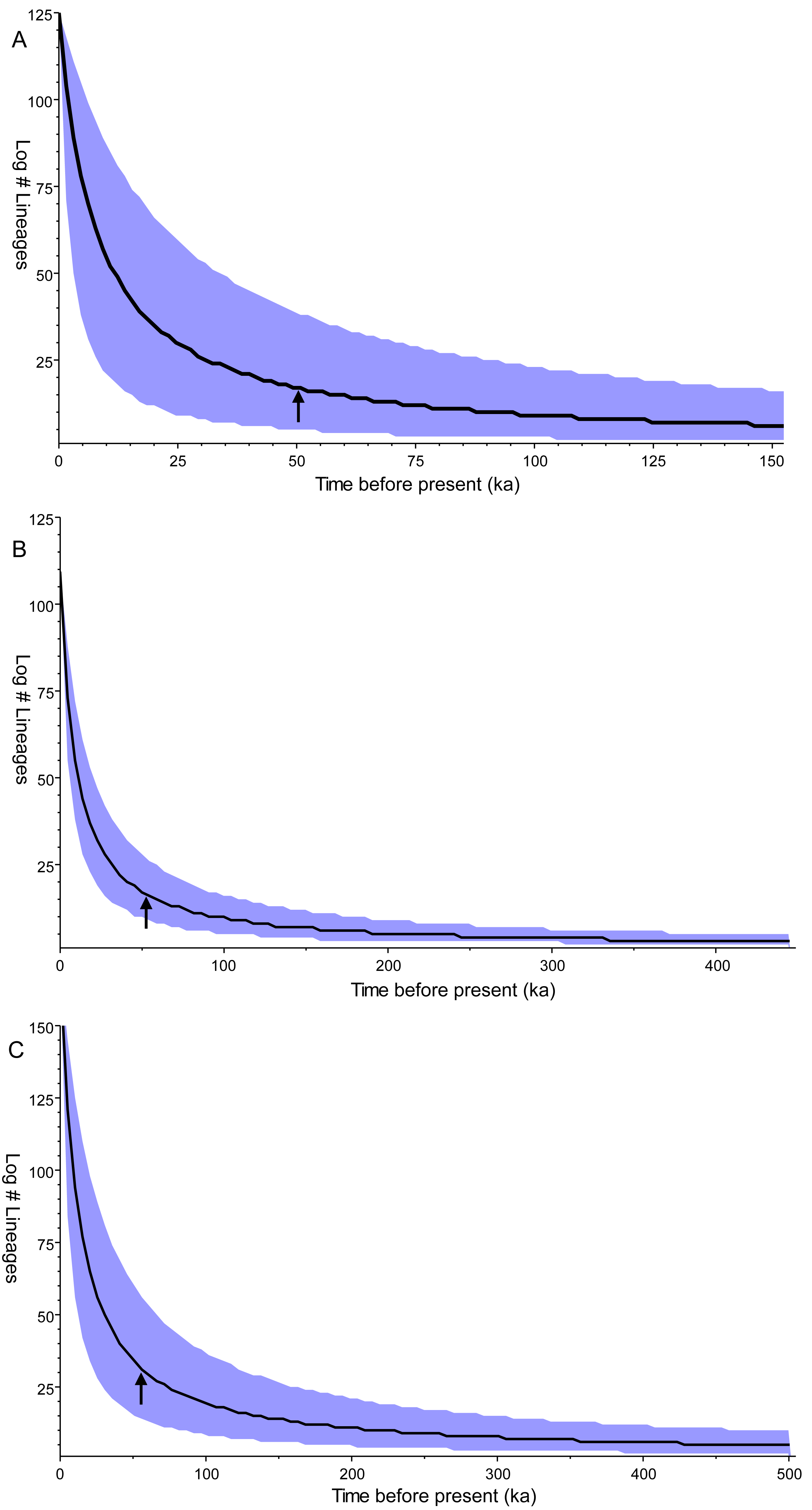

Supplement: Figure S3 — Lineage through time plots for Clematis sibirica based on cpDNA (A) and nrITS (B) datasets and C. songorica based on cpDNA (C) dataset. Arrows indicate the occurrence of interglacial periods in the study area. (TIF) [file pone.0061954.s003.tif]
