# Supplementary material for: Retreating or Standing: Responses of Forest Species and Steppe Species to Climate Change in Arid Eastern Central Asia
Source: PLoS One. 2013 Apr 15;8(4):e61954. doi: 10.1371/journal.pone.0061954 (PMC3626637; doi:10.1371/journal.pone.0061954)
Supplement: Table S1 — Details of sampling localities for the 28 Clematis sibirica and 38 Clematis songorica populations studied. Population codes are numbered consecutively, as shown on the map in Figure 1. Number of individuals and genotype distribution in the cpDNA (cpN ind) and nrITS (nrN ind) analysis were shown. (DOC) [file pone.0061954.s004.doc]

Table S1. Details of sampling localities for the 28 *Clematis sibirica* and 38 *Clematis songorica* populations studied. Population codes are numbered consecutively, as shown on the map in Fig. 1. Number of individuals and genotype distribution in the cpDNA (cp*N*_ind_) and nrITS (nr*N*_ind_) analysis were shown.

| Code | Population | Latitude ⁄ longitude | cp*N*_ind_ | Chlorotypes | nr*N*_ind_ | Ribotypes |
| --- | --- | --- | --- | --- | --- | --- |
| *Clematis sibirica* | |  | 125 |  | 109 |  |
| 1 | FBL | 45°12’N/81°46’E | 5 | H1, H2 | 4 | N1 |
| 2 | FTL | 45°47’N/82°59’E | 5 | H1 | 5 | N1 |
| 3 | FHF | 46°59’N/85°50’E | 5 | H1, H2 | 5 | N1 |
| 4 | FJMN | 47°9’N/86°9’E | 5 | H1 | 3 | N1 |
| 5 | FHBH | 48°19’N/86°45’E | 3 | H1, H2 | 4 | N1 |
| 6 | FBEJB | 48°30’N/87°8’E | 5 | H1, H2 | 5 | N1 |
| 7 | FBEJA | 48°25’N/87°12’E | 5 | H1, H2 | 5 | N1 |
| 8 | FALT | 47°59’N/88°14’E | 2 | H1 | 2 | N1 |
| 9 | FFH | 47°42’N/89°1’E | 2 | H1, H4 | 4 | N1 |
| 10 | FFY | 47°11’N/89°52’E | 5 | H1, H4 | 5 | N1 |
| 11 | FQH | 46°56’N/90°16’E | 5 | H1 | 4 | N1 |
| 12 | FYW | 43°24’N/93°59’E | 5 | H1, H3 | 5 | N1 |
| 13 | FHM | 43°17’N/93°48’E | 5 | H1, H4, H5 | 4 | N1 |
| 14 | FBLK | 43°33’N/93°0’E | 5 | H1 | 5 | N1 |
| 15 | FQT | 43°33’8N/89°44’E | 4 | H1, H2 | 5 | N1 |
| 16 | FFK | 43°54’N/88°7’E | 5 | H1, H6 | 4 | N1, N2 |
| 17 | FWLMQ | 43°14’N/87°9’E | 5 | H1, H4, H6 | 4 | N1 |
| 18 | FCJ | 43°29’N/86°58’E | 5 | H6 | 3 | N1, N5 |
| 19 | FMNS | 43°49’N/86°12’E | 5 | H6, H7 | 5 | N2, N3, N4 |
| 20 | FSW | 43°53’N/85°24’E | 5 | H1, H6 | 5 | N1, N3 |
| 21 | FWUS | 44°9’N/84°20’E | 5 | H6 | 5 | N2, N3 |
| 22 | FXY | 43°14’N/84°38’E | 5 | H6, H7 | 5 | N2, N10, N11 |
| 23 | FJH | 44°20’N/83°7’E | 5 | H6 | 3 | N6, N7 |
| 24 | FHC | 44°27’N/81°8’E | 3 | H6, H7 | 1 | N8 |
| 25 | FTKS | 42°56’N/81°46’E | 4 | H6, H7 | 2 | N2, N9 |
| 26 | FCBCE | 43°29’N/81°6’E | 2 | H6 | 1 | N3 |
| 27 | FZS | 42°41’N/80°46’E | 5 | H6, H7 | 4 | N2 |
| 28 | FWENS | 41°49’N/80°41’E | 5 | H6, H7, H8 | 2 | N2 |
| *Clematis songorica* | |  | 169 |  | 116 |  |
| 29 | SBL | 45°4’N/81°50’E | 6 | h2 | 5 | A1, A1+A3 |
| 30 | STLA | 45°36’N/82°41’E | 5 | h2, h10 | 5 | A1+A2 |
| 31 | STLB | 45°44’N/83°3’E | 3 | h2 | 4 | A1+A5, A1+A3 |
| 32 | STLC | 45°50’N/83°20’E | 5 | h2 | 4 | A1, A1+A5, A7 |
| 33 | SEM | 46°46’N/83°46’E | 5 | h8 | 4 | A1 |
| 34 | SHF | 46°37’N/85°39’E | 5 | h2, h8 | 3 | A1+A3 |
| 35 | SJMNB | 47°12’N/86°7’E | 5 | h3, h8 | 3 | A1, A1+A5 |
| 36 | SJMNA | 47°8’N/86°36’E | 4 | h8 | 5 | A1+A5, A1+A3, A6 |
| 37 | SALT | 47°55’N/88°8’E | 4 | h8 | 2 | A1+A5 |
| 38 | SFY | 46°58’N/89°42’E | 5 | h1 | 5 | A1 |
| 39 | SQH | 46°29’N/90°9’E | 6 | h1 | 3 | A1, A1+A5 |
| 40 | SYW | 43°25’N/94°2’E | 4 | h2 | 1 | A2 |
| 41 | SHM | 43°8’N/93°48’E | 5 | h2 | 4 | A2 |
| 42 | SBLK | 43°35’N/93°1’E | 5 | h2 | 3 | A1, A2 |
| 43 | SML | 43°57’N/89°57’E | 5 | h1 | 1 | A1 |
| 44 | SQT | 43°54’N/89°40’E | 5 | h1 | 3 | A1+A5 |
| 45 | SJMSE | 43°55’N/89°6’E | 4 | h1 | 4 | A5, A1+A5 |
| 46 | SFK | 44°5’N/88°38’E | 5 | h1 | 3 | A5, A1+A5 |
| 47 | SSS | 42°54’N/90°16’E | 3 | h1, h8 | 3 | A5, A1+A5 |
| 48 | STLF | 43°8’N/88°57’E | 4 | h1 | 2 | A1, A1+A5 |
| 49 | SDBC | 43°22’N/88°15’E | 5 | h1 | 2 | A1+A5 |
| 50 | SWLMQ | 43°29’N/87°17’E | 5 | h1 | 5 | A1, A1+A5 |
| 51 | SCJ | 43°30’N/86°59’E | 5 | h1 | 5 | A1, A1+A5, A1+A2 |
| 52 | SMNS | 43°55’N/86°16’E | 5 | h1 | 3 | A1+A5 |
| 53 | SSW | 43°55’N/85°24’E | 1 | h6 | 1 | A1+A3 |
| 54 | SWUS | 44°13’N/84°23’E | 4 | h2, h9 | 3 | A1+A5, A1+A3 |
| 55 | SJH | 44°23’N/83°4’E | 5 | h2 | 3 | A3, A1+A5 |
| 56 | SCBCE | 43°43’N/81°5’E | 5 | h2 | 2 | A1, A1+A5 |
| 57 | STKS | 43°2’N/81°47’E | 5 | h2 | 3 | A1 |
| 58 | SHS | 42°15’N/87°16’E | 5 | h9 | 4 | A7, A8 |
| 59 | SHJ | 42°45’N/86°18’E | 5 | h9 | 4 | A1+A5 |
| 60 | SLT | 42°0’N/85°4’E | 4 | h2, h8, h9 | 1 | A1 |
| 61 | SKC | 41°42’N/83°1’E | 4 | h2, h4 | 4 | A1, A4 |
| 62 | SBC | 41°59’N/81°30’E | 5 | h2, h3, h7 | 3 | A1 |
| 63 | SWENS | 41°44’N/80°42’E | 4 | h1, h3, h8 | 2 | A1 |
| 64 | SWQB | 39°42’N/73°58’E | 3 | h2, h3 | 1 | A1 |
| 65 | SWQA | 39°42’N/75°16’E | 5 | h3 | 2 | A1 |
| 66 | SAKT | 39°18’N/75°32’E | 1 | h5 | 1 | A1 |
