# Supplementary material for: Retreating or Standing: Responses of Forest Species and Steppe Species to Climate Change in Arid Eastern Central Asia
Source: PLoS One. 2013 Apr 15;8(4):e61954. doi: 10.1371/journal.pone.0061954 (PMC3626637; doi:10.1371/journal.pone.0061954)
Supplement: Table S2 — Variable sites of each genotype for the cpDNA fragment ( psb A- trn H) and the nrITS region in Clematis sibirca and C. songorica . (DOC) [file pone.0061954.s005.doc]

Table S2. Variable sites of each genotype for the cpDNA fragment (*psb*A-*trn*H) and the nrITS region in *Clematis sibirca* and *C. songorica*.

| *Clematis sibirica* | Variable sites | | | | | | | | | | | | | | | |
| --- | --- | --- | --- | --- | --- | --- | --- | --- | --- | --- | --- | --- | --- | --- | --- | --- |
|  | 1 | 1 | 2 | 2 | 2 |  |  | 2 | 2 | 2 | 2 |  |  | 2 | 2 | 3 |
|  | 1 | 9 | 0 | 1 | 1 |  |  | 1 | 1 | 1 | 1 |  |  | 2 | 6 | 4 |
| Chlorotype | 6 | 9 | 0 | 0 | 1 |  |  | 4 | 5 | 6 | 7 |  |  | 0 | 0 | 9 |
| H1 | G | – | – | C | A | A | T | T | G | A | – | – | – | – | C | T |
| H2 | G | – | – | C | A | A | T | T | G | A | – | – | – | – | C | – |
| H3 | G | T | T | C | A | A | T | T | G | A | – | – | – | – | C | T |
| H4 | G | T | T | A | A | A | T | T | A | A | – | – | – | – | T | T |
| H5 | A | T | T | A | A | A | T | T | – | – | T | T | T | T | T | T |
| H6 | G | T | T | A | – | – | – | – | – | – | – | – | – | – | T | T |
| H7 | G | T | T | A | – | – | – | – | – | – | – | – | – | – | T | – |
| H8 | A | T | T | A | – | – | – | – | – | – | – | – | – | – | T | T |

–, indel.

| *Clematis sibirica* | Variable sites | | | | | | | | | | | | | | | | | | |
| --- | --- | --- | --- | --- | --- | --- | --- | --- | --- | --- | --- | --- | --- | --- | --- | --- | --- | --- | --- |
|  |  |  |  |  |  |  |  | 1 | 1 | 1 | 3 | 3 | 4 | 4 | 4 | 4 | 5 | 5 | 5 |
|  | 5 | 6 | 6 | 6 |  |  | 7 | 6 | 6 | 8 | 3 | 9 | 3 | 3 | 4 | 9 | 0 | 5 | 6 |
| Ribotype | 9 | 0 | 7 | 9 |  |  | 2 | 4 | 9 | 0 | 1 | 7 | 6 | 9 | 8 | 8 | 9 | 0 | 1 |
| N1 | G | – | – | – | – | – | – | A | A | T | C | A | T | T | G | C | T | C | G |
| N2 | C | C | C | C | C | G | G | G | G | T | T | C | T | C | G | G | C | T | A |
| N3 | C | C | C | C | C | G | G | A | A | C | T | C | C | C | G | G | C | T | A |
| N4 | C | C | C | C | C | G | G | G | G | C | T | C | C | C | G | G | C | T | A |
| N5 | G | – | – | – | – | – | – | G | G | T | T | A | T | C | G | C | T | T | A |
| N6 | C | C | C | C | C | G | G | G | G | T | C | C | T | C | G | G | C | T | A |
| N7 | C | C | C | C | C | G | G | A | A | C | T | C | T | C | G | G | C | T | A |
| N8 | C | C | C | C | C | G | G | G | G | T | C | C | T | C | G | G | C | T | G |
| N9 | C | C | C | C | C | G | G | A | G | T | T | C | T | C | A | G | C | T | A |
| N10 | C | C | C | C | C | G | G | A | G | T | T | C | C | C | G | G | C | T | A |
| N11 | C | C | C | C | C | G | G | G | G | T | T | C | C | C | G | G | C | T | A |

–, indel.

| *Clematis songorica* | | | Variable sites | | | | | | | | | | | | | | | | | | | | | | | | | | | | |
| --- | --- | --- | --- | --- | --- | --- | --- | --- | --- | --- | --- | --- | --- | --- | --- | --- | --- | --- | --- | --- | --- | --- | --- | --- | --- | --- | --- | --- | --- | --- | --- |
|  | 1 | 1 |  | 2 | 2 | 2 | 2 |  |  |  |  |  |  | 2 | 2 | 3 | 3 |  |  |  |  |  |  | 3 | 3 |  |  |  | 3 | 3 | 3 |
|  | 8 | 9 |  | 0 | 0 | 1 | 1 |  |  |  |  |  |  | 1 | 4 | 0 | 0 |  |  |  |  |  |  | 1 | 2 |  |  |  | 2 | 3 | 4 |
| Chlorotype | 9 | 8 |  | 0 | 9 | 0 | 1 |  |  |  |  |  |  | 8 | 7 | 4 | 5 |  |  |  |  |  |  | 2 | 5 |  |  |  | 9 | 6 | 4 |
| h1 | C | T | T | T | T | A | – | – | – | – | – | – | – | – | G | T | – | – | – | – | – | – | – | – | – | – | – | – | – | C | T |
| h2 | C | – | – | – | A | A | – | – | – | – | – | – | – | – | A | T | A | A | A | C | C | A | A | T | – | – | – | – | – | C | T |
| h3 | C | T | T | T | T | A | A | A | T | T | T | G | A | T | G | T | – | – | – | – | – | – | – | – | – | – | – | – | – | A | G |
| h4 | C | T | T | T | A | A | – | – | – | – | – | – | – | – | A | T | A | A | A | C | C | A | A | T | – | – | – | – | – | C | T |
| h5 | C | T | T | T | T | T | A | A | T | T | T | G | A | T | G | T | – | – | – | – | – | – | – | – | – | – | – | – | – | A | G |
| h6 | C | – | – | – | A | A | – | – | – | – | – | – | – | – | A | T | A | A | A | C | C | A | A | T | A | A | T | A | T | C | T |
| h7 | C | T | T | T | T | A | – | – | – | – | – | – | – | – | G | T | – | – | – | – | – | – | – | – | – | – | – | – | – | A | G |
| h8 | A | – | – | – | A | A | – | – | – | – | – | – | – | – | A | T | A | A | A | C | C | A | A | T | – | – | – | – | – | C | T |
| h9 | C | – | – | – | C | A | – | – | – | – | – | – | – | – | A | T | A | A | A | C | C | A | A | T | – | – | – | – | – | C | T |
| h10 | C | – | – | – | T | C | – | – | – | – | – | – | – | – | G | – | – | – | – | – | – | – | – | T | – | – | – | – | – | A | T |

–, indel.

| *Clematis songorica* | Variable sites | | | | | | |
| --- | --- | --- | --- | --- | --- | --- | --- |
|  | 1 | 3 | 3 | 3 | 3 | 3 | 4 |
|  | 4 | 0 | 1 | 4 | 8 | 9 | 7 |
|  | 6 | 4 | 7 | 0 | 3 | 7 | 3 |
| Allele of nrITS |  |  |  |  |  |  |  |
| A1 | T | C | G | C | T | G | C |
| A2 | T | C | G | C | C | G | C |
| A3 | C | C | G | A | C | G | C |
| A4 | T | C | G | C | T | A | C |
| A5 | C | C | G | C | C | G | C |
| A6 | T | C | G | C | T | G | T |
| A7 | T | C | A | C | T | G | C |
| A8 | T | A | G | C | T | G | C |
| Heterozygote genotypes |  |  |  |  |  |  |  |
| A1+A5 | Y | C | G | C | Y | G | C |
| A1+A2 | T | C | G | C | Y | G | C |
| A1+A3 | Y | C | G | M | Y | G | C |
